# Difficulties in reporting purpose and dosage of prescribed medications are associated with poor cognition and depression

**DOI:** 10.1371/journal.pone.0251374

**Published:** 2021-05-13

**Authors:** Hannah M. Zipprich, Tino Prell

**Affiliations:** 1 Department of Neurology, Jena University Hospital, Jena, Germany; 2 Center for Healthy Ageing, Jena University Hospital, Jena, Germany; University of Toronto, CANADA

## Abstract

Knowledge on prescribed medication is important for medication adherence. We determined the presence of cognitive impairment in neurological patients who report not to know reasons and dosages of their medication. Data from 350 patients were collected: sociodemographic data, German Stendal Adherence to Medication Score (SAMS), Montreal Cognitive Assessment (MoCA), and Beck Depression Inventory-II (BDI-II). Eighty-eight (29.0%) patients did not know the reasons for taking their prescribed medication and 83 (27.4%) did not know the doses. Sixty-three (20.8%) knew neither reasons nor dosage. The latter were characterized by higher nonadherence, higher number of prescribed medication per day, lower MoCA, higher BDI, and had more often a lower education level compared with patients who knew the reasons. The MANOVA revealed a significant multivariate effect for not knowing the reasons and not knowing the dosages of medication on MoCA and BDI. Significant univariate effects for not knowing reasons were found for depressive mood, but not for cognitive performance. Significant univariate effects for not knowing dosages were found for cognitive performance, but not for depressive mood. Inaccurate medication reporting is not solely associated with cognitive problems, but also with depression, which has to be taken into account in daily practice and research.

## Introduction

Medication adherence is a crucial part of chronic disease management [1]. Among others, health literacy might contribute to medication adherence [2]. Health literacy is “the degree to which individuals have the capacity to obtain, process, and understand basic health information and services needed to make appropriate health decisions” [3–5]. Inadequate health literacy is common and has been associated with poorer health, higher medical expenses, less than optimal use of preventive health services, increased hospitalization, and medication nonadherence [6]. In particular for medication adherence, it is essential that people have sufficient knowledge about their prescribed medication. People with good knowledge about their medication can be intentionally nonadherent, while people with poor knowledge are more likely to err due to misunderstanding instructions (unintentional nonadherence) [7,8]. Therefore, health literacy and medication adherence depend on a range of cognitive abilities [9,10]. Hence, for example, the pill questionnaire has been proposed as a screening instrument for assessing decline in cognitive patients with Parkinson´s disease (with low sensitivity) [11,12]. Given the association between knowledge about medication, nonadherence, and cognition, we aimed to answer the following question: If patients report not to know the reasons for their medication nor the dosages, can it be concluded that they have cognitive impairment?.

## Materials and methods

The study was approved by the local ethics committee of the Jena University Hospital (5290-10/17) and written informed consent was obtained from all patients. Data from 350 patients was collected either during their visit to the outpatient clinic or during their stay in the neurological ward in the Department of Neurology at the Jena University Hospital, which includes age, gender, marital status (single, divorced, widowed, or married), education level (high, German Abitur or University; medium, German Realschule; low, German Hauptschule or no school), and number of drugs per day. Adherence was assessed using the German Stendal Adherence to Medication Score (SAMS) that includes 18 questions forming a cumulative scale [13]. The sum of the scores ranged from 0 (fully adherent) to 72 (fully nonadherent). The whole copy of SAMS is available online (CC BY NC 3.0 license; https://data.mendeley.com/datasets/ny2krr3vgg/1) [14]. In SAMS, the first question is “Do you know the reason for taking your medication?” (for all, for most, for half, for some or for none). The second question is “Do you know the dosages of your medication?” (for all, for most, for half, for some or for none). The patients were then categorized into those who know/do not know the reasons for their prescribed medication and those who know/do not know the dosages of their prescribed medication. Cognition was assessed using the Montreal Cognitive Assessment (MoCA) and a cutoff of 19 points was used to exclude patients with significant cognitive decline [15]. Depression was assessed using the Beck Depression Inventory-II (BDI-II).

SPSS statistical computer package (version 25.0; IBM Corporation, USA) was used for all statistical analyses. Values are expressed as mean and standard deviation (SD) when normally distributed and otherwise as median and interquartile range (IQR). Categorical variables are expressed as numbers or percentages. Descriptive statistics was used to describe the cohort. Group comparisons were performed with analysis of variance for normally distributed data and Kruskal–Wallis test for non-normally distributed data (with Dunn–Bonferroni correction). A MANOVA was conducted to examine whether “not to know reasons” and “not to know dosages” (independent variables) are associated with (1) cognition (MoCA) and (2) depressive mood (BDI-II). MoCA and BDI were entered into MANOVA as dependent variables. A MANCOVA was also conducted to control for potential confounds, including age, number of drugs per day, and education level.

Logistic regression with “knowing reasons for prescribed medication” (yes/no) as dependent variable and age, education level, number of drugs per day, BDI, and MoCA as independent variables was performed (stepwise forward selection).

## Results

**Fig 1:**
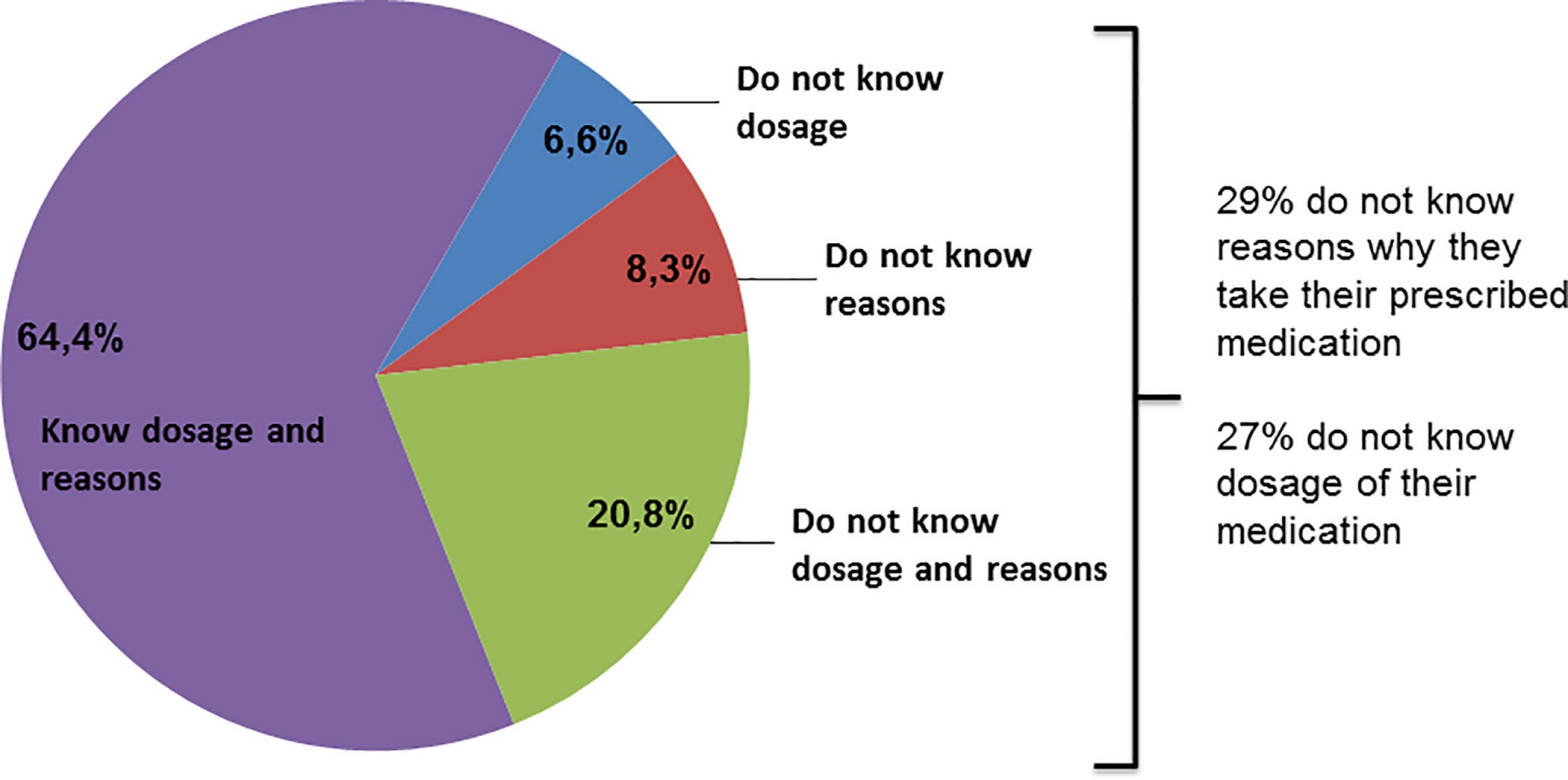
Knowledge about medication.

Overall, 350 patients were enrolled into the study. After exclusion of 47 patients (MoCA < 19) 303 patients were included into the following analyses. Detailed epidemiological characteristics are presented in Table 1. Eighty-eight (29.0%) patients reported not to know the reasons for taking their prescribed medication and 83 (27.4%) reported not to know the doses. Sixty-three (20.8%) knew neither reasons nor dosages (Fig 1). Patients who did not know the dosages or reasons for taking their prescribed medication were characterized by higher nonadherence, higher number of prescribed medication per day, poorer cognition (lower MoCA), higher depressive burden (higher BDI) and a higher proportion of people with lower education level compared with people who know the reasons (Table 2).

**Table 1:** Clinical and epidemiological characteristics.

		Mean	SD
Age		68.3	9.7
Medication per day		5.4	3.3
Montreal Cognitive Assessment total		24.0	3.1
Beck Depression Inventory total		10.9	8.5
		**N**	**%**
Gender	Female	123	40.6
	Male	180	59.4
Marital status	Missing data	4	1.3
	Single	23	7.6
	Married	200	66.0
	Divorced, widowed or separated	76	25.1
Living situation	Missing data	51	16.8
	Single	68	22.4
	With others	184	60.7
Education level	Missing data	8	2.6
	High	103	34.0
	Medium	104	34.3
	Low	88	29.0

**Table 2:** Group comparison.

		Do not know dosage		Do not know reasons		Do not know dosage and reasons		Know dosage and reasons	
		Mean	SD	Mean	SD	Mean	SD	Mean	SD
**Age**		66.2	9.8	70.7	8.5	69.1	8.8	67.9	10.1
**Medication per day**		5.7_a,b_	2.9	8.1_a_	4.1	6.4_a_	3.2	4.8_b_	3.1
**SAMS total**		9.4 _a_	8.0	9.4 _a_	6.2	15.0_b_	10.0	4.4_c_	6.1
**MoCA**		23.0 _a,b_	3.3	24.0 _a,b_	3.2	22.4 _a_	3.2	24.5 _b_	2.9
**BDI**		10.8 _a,b_	8.8	14.5 _a_	8.0	13.8 _a_	10.5	9.4 _b_	7.4
		**n**	**%**	**n**	**%**	**n**	**%**	**n**	**%**
**Gender**	Female	5	4.1	10	8.1	18	14.6	90	73.2
	Male	15	8.3	15	8.3	45	25.0	105	58.3
**Education**	High	7	6.8	8	7.8	18	17.5	70	68.0
	Middle	6	5.8	9	8.7	15	14.4	74	71.2
	Low	6	6.8	7	8.0	27	30.7	48	54.5

Values in the same row and sub-table where the subscript is not identical differ greatly at *p* < .05 in the two-sided equality test for column averages. Using the Bonferroni correction, the tests are adapted to all paired comparisons within a row of the innermost sub-table.

BDI, Beck Depression Inventory; MoCA, Montreal Cognitive Assessment; SAMS, Stendal Adherence with Medication Score; SD, standard deviation.

The MANOVA revealed a significant multivariate main effect for not knowing the reasons (Wilks’ λ = 0.97, *p* = .009, partial η^2^ = .031) and for not knowing the dosages (Wilks’ λ = 0.97, *p* = .009, partial η^2^ = .031) of medication on cognitive performance (MoCA) and mood (BDI). The association between not knowing the reasons and not knowing the dosages was not significant (Wilks’ λ = 0.99, *p* = .758, partial η^2^ = .002). Significant univariate main effects for not knowing the reasons were found for depressive mood (*p* = .004), but not for cognitive performance (*p* = .262). Significant univariate main effects for not knowing dosages were found for cognitive performance (*p* = .002), but not for depressive mood (*p* = .807).

To examine whether these findings could be accounted for by other medical covariates, a MANCOVA was conducted. Here, age (Wilks’ λ = 0.95, *p* = .001, partial η^2^ = .05) and education level (Wilks’ λ = 0.96, *p* = .025, partial η^2^ = .02) were also found statistically significant while number of drugs per day was not (*p* = .29). In addition, the results did not change after controlling for these variables, as there was a significant main effect of not knowing the reasons (Wilks’ λ = .97, *p* = .045, partial η^2^ = .024) and not knowing dosages (Wilks’ λ = .97, *p* = .002, partial η^2^ = .05).

In the logistic regression “not to know reasons for prescribed medication” was associated with BDI (OR = 1.06, *p* = .001, 95%CI 1.03–1.1), MoCA (OR = 0.85, *p* = .001, 95%CI 0.77–0.93), and total number of drugs per day (OR = 1.18, *p* < .001, 95%CI 1.08–1.27) (Nagelkerke R2 = 0.22, Hosmer–Lemeshow = 0.56). Not to know dosages of prescribed medication was associated with BDI (OR = 1.04, *p* = .036, 95%CI 1.002–1.075) and MoCA (OR = 0.75, *p* < .001, 95%CI 0.71–0.86) (Nagelkerke R2 = 0.16, Hosmer–Lemeshow = 0.14).

## Discussion

This exploratory study was based on the question of what can be concluded in terms of cognition when patients report not to know the reasons for or dosages of their medication. About 30% of all patients indicated that they did not know the reasons for or dosage of their medication. This is in line with a previous interview-based study of patients from 6 US primary care clinics where 30% of patients could not name at least 1 of their medications and 19% did not know their purpose [6]. As expected, these patients in our cohort showed generally poorer adherence (higher SAMS total score). In addition, they were characterized by poorer cognitive performance and more depression. In the univariate analysis, we found that the number of drugs, MoCA, BDI, and educational level seem to play a role for less knowledge on reasons and dosages of medication. Our multivariable analysis and logistic regression showed that not knowing the reasons for medication is more likely associated with a depressive disorder. In contrast, not knowing the dosage of medication tended to indicate impaired cognition. To the best of our knowledge, this is a novel finding because inaccurate medication reporting was so far only found to be associated with cognitive function and not with mood disorders. Our study indicates that depression is a relevant cofactor for inaccurate medication reporting and should be taken into account when measures such as the pills questionnaire were used.

Given that medication health literacy is influenced by cognitive function [16–18], it is obvious that deficits in different cognitive domains and the associated restrictions in planning, organizing, and taking medications are relevant for adherence [1,19–21]. However, the available data is not as clear as one would assume. Although some studies reported no association between medication adherence and cognitive impairment, other studies found that cognitive impairment was associated with increased or decreased non-adherence [22]. However, for depression, depressive symptoms are shown to be associated with nonadherence and it has even been postulated that suddenly occurring nonadherence could indicate depression as a cause [23–26].

This study has several limitations. First, the study is limited to hospitalized patients; hence no statement can be made about community-dwelling people. In addition, health literacy was not comprehensively covered by questionnaire as it was not the primary end point of the study. Instead, the lack of knowledge on reasons and dosages of drugs was assumed to be an expression of lower health literacy. Although the relationship between cognition and adherence is not yet fully understood, we excluded patients with severe cognitive impairment from our study. We assumed that severe dementia could possibly influence the validity of self-report questionnaires. Therefore, the results of our exploratory study are limited to hospitalized patients without dementia and further confirmatory studies are necessary to investigate the association between specific lack of knowledge, cognition, and mood disorders.

## Conclusion

Impaired knowledge about reasons and dosage of prescribed medication is a common indicator of non-adherence and low health literacy. Our data show that when people report not knowing about their medications, not only cognitive disorders should be considered as a cause, but also depressive disorders. Questions about knowledge of medications are therefore of limited use as screening for cognitive deficits in clinical practice.

## Supporting information

S1 Data (XLSX)
